# E-Mail-Related Problem-Solving Behaviors Across Age Groups: An Analysis of Log File Data

**DOI:** 10.1093/geroni/igaa057.1323

**Published:** 2020-12-16

**Authors:** Taka Yamashita, Leah Saal, Roberto Millar, Shalini Sahoo, Phyllis Cummins

**Affiliations:** 1 University of Maryland, Baltimore, Maryland, United States; 2 Loyola University, Baltimore, Maryland, United States; 3 Graduate Student, Baltimore, Maryland, United States; 4 University of Maryland, Baltimore, Elkridge, Maryland, United States; 5 Miami University, Oxford, Ohio, United States

## Abstract

Email is one of the most common and useful online communication tools. However, older adults tend to have difficulties fully taking advantage of email. Organizing the information in the email software environment and sending a message to selected recipients are examples of common email-related problem-solving. To date, little data are available to understand the email-related problem-solving behaviors of older adults. Nationally representative survey data and log-file data of the U.S. adults (n = 1,341) are derived from the 2012 Program for International Assessment of Adult Competencies (PIAAC), which provides computer-based assessment data on problem-solving skills. The PIAAC respondents used the computer device and solved the problems in the simulated email environment. Descriptive summary showed that those 55 years and older took longer (169 seconds), referred to the help menu (15%) and used the cancel button (26%) more often than younger age groups (e.g., age 25-34; 103 seconds, 3% and 17%, respectively) in one of the tasks. Additionally, binary logistic regression showed that taking longer time (odds-ratio = 0.99, p < 0.05) and using the help menu (odds-ratio = 0.85, p < 0.05) were associated with the incorrect answer to the email problem-solving, although the findings varied across different types of problems. These unique findings from the combination of survey and log-file data analyses suggested that some older adults may benefit from the training for common email-related problems rather than teaching themselves. Detailed descriptions of computer-based assessment log file data and other results are also evaluated in this study.

